# Electroshock synthesis of a bifunctional nonprecious multi‐element alloy for alkaline hydrogen oxidation and evolution

**DOI:** 10.1002/EXP.20220024

**Published:** 2022-11-24

**Authors:** Lijie Du, Hu Xiong, Hongcheng Lu, Li‐Ming Yang, Rong‐Zhen Liao, Bao Yu Xia, Bo You

**Affiliations:** ^1^ Key Laboratory of Material Chemistry for Energy Conversion and Storage, Ministry of Education, Hubei Key Laboratory of Material Chemistry and Service Failure, School of Chemistry and Chemical Engineering Huazhong University of Science and Technology (HUST) Wuhan Hubei China

**Keywords:** electrocatalysis, electroshock, H_2_ evolution, H_2_ oxidation, multi‐element alloy

## Abstract

The design and production of active, durable, and nonprecious electrocatalysts toward alkaline hydrogen oxidation and evolution reactions (HOR/HER) are extremely appealing for the implementation of hydrogen economy, but remain challenging. Here, we report a facile electric shock synthesis of an efficient, stable, and inexpensive NiCoCuMoW multi‐element alloy on Ni foam (NiCoCuMoW) as a bifunctional electrocatalyst for both HOR and HER. For the HOR, the current density of NiCoCuMoW could reach ∼11.2 mA cm^–2^ when the overpotential is 100 mV, higher than that for commercial Pt/C (∼7.2 mA cm^–2^) and control alloy samples with less elements, along with superior CO tolerance. Moreover, for the HER, the overpotential at 10 mA cm^−2^ for NiCoCuMoW is only 21 mV, along with a Tafel slope of low to 63.7 mV dec^−1^, rivaling the commercial Pt/C as well (35 mV and 109.7 mV dec^−1^). Density functional theory calculations indicate that alloying Ni, Co, Cu, Mo, and W can tune the electronic structure of individual metals and provide multiple active sites to optimize the hydrogen and hydroxyl intermediates adsorption, collaboratively resulting in enhanced electrocatalytic activity.

## Graphical abstract

A facile electric shock method is used to synthesize an efficient, stable, and inexpensive NiCoCuMoW bifunctional alloy electrocatalyst for both hydrogen oxidation reaction (HOR) and hydrogen evolution reaction (HER). Combined experiments and theory calculations reveal that the multi‐component synergy of different elements and plentiful active sites render the NiCoCuMoW superior HOR/HER performance, rivaling the Pt/C benchmark and most reported nonprecious catalysts.

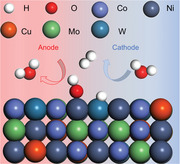

## 1 INTRODUCTION

To address the energy crisis due to the persistent consumption of unrenewable fossil fuel and the associated detrimental environment issues, a new prototype called hydrogen economy has been proposed to replace the prevailing hydrocarbon economy long ago.^[^ ^1^, ^2^ ^]^ It converts the transient and intermittent renewable energy from the sun and wind into the chemical energy of hydrogen molecule (H_2_) via (photo)electrochemical water splitting, and then the stored energy can be released on demand through H_2_‐O_2_ fuel cells.^[^ ^3^, ^4^ ^]^ During the hydrogen cycling, no pollutants such as CO_2_ and NO*_x_* are produced.^[^ ^5^ ^]^ Despite the promising prospects of hydrogen economy, its commercial implementation largely relies on the efficient H_2_ utilization and production, both of which require economical, active, and durable electrocatalysts to promote the sluggish kinetics of the involved hydrogen oxidation and evolution reactions (HOR and HER).^[^ ^6^, ^7^ ^]^ Although proton exchange membrane (PEM)‐based fuel cells (PEMFCs) and water electrolyzers (PEMWEs) have witnessed great progress in the past years,^[^ ^8^, ^9^ ^]^ the acidic media constrains the candidate pool of nonprecious electrocatalysts which means that only platinum group metals (PGM) including Pt, Ru, and Ir can be used.^[^ ^10^, ^11^ ^]^ Their high cost and dearth of PGM have restricted the commodification. In response, anion‐exchange membrane (AEM) based energy transformation technologies have received increasing research interest because their alkaline media are friendly to PGM‐free electrocatalysts.^[^ ^12^, ^13^, ^14^ ^]^ However, alkaline medium is relatively critical for both HOR and HER electrodes. For example, even for PGM‐based electrocatalysts, their catalytic activities of HOR and HER would decrease by dozens of times when changing the electrolyte from acid to alkaline media.^[^ ^15^ ^]^ Many efforts have been devoted to developing efficient and cost‐effective electrocatalysts for alkaline HOR and HER. Recently, Ni_3_N/C nanoparticles,^[^ ^16^ ^]^ Ni/CeO_2_,^[^ ^17^ ^]^ CoNiMo,^[^ ^18^ ^]^ Ni/N–doped carbon nanotubes,^[^ ^19^ ^]^ Cr‐Ni,^[^ ^20^ ^]^ MoNi_4_,^[^ ^21^ ^]^ Ni_5.2_WCu_2.2_,^[^ ^22^ ^]^ etc. have shown promising HOR activity, and nonprecious metal‐based sulfides,^[^ ^23^ ^]^ carbides,^[^ ^24^ ^]^ phosphides,^[^ ^25^ ^]^ and selenides^[^ ^26^ ^]^ were reported to be active for the HER. However, their alkaline HOR/HER activities remain lower than the commercially available Pt catalysts because of the difficulty to simultaneously optimize the binding with critical intermediates like H, OH species, and so on.^[^ ^27^ ^]^ It's thus highly desirable to explore effective nonprecious bifunctional electrocatalysts that are active for both HOR and HER in alkaline solutions.

Alloying that combines different metals with various properties together has been proven to be prominent in altering the electronic structures of the alloyed metals, leading to a synergetic interaction that tunes the adsorption energies of reactive intermediates for enhanced catalytic reactivity.^[^ ^28^, ^29^ ^]^ Specifically, multi‐element alloy (MEA) has drawn increasing interest in the fields of energy electrocatalysis due to its multiple advantages.^[^ ^30^, ^31^ ^]^ First, the multi‐component nature of MEA provides multiple sites to adsorb different intermediates respectively.^[^ ^32^ ^]^ Second, the electronic structures of active sites which determine the catalytic performances can be finely tuned by the surrounding atoms.^[^ ^33^, ^34^, ^35^, ^36^ ^]^ Nowadays, MEA has been reported to catalyze HER,^[^ ^37^, ^38^ ^]^ oxygen evolution,^[^ ^39^, ^40^ ^]^ oxygen reduction,^[^ ^41^ ^]^ and CO_2_ reduction.^[^ ^42^ ^]^ However, to our knowledge, there are few relevant reports investigating the application of non‐precious metal based MEA for alkaline HOR. Moreover, traditional methods for the synthesis of MEA like arc melting, high‐temperature thermal shock (∼2000 K) and high‐energy mechanical balling often need complex procedures and/or harsh physiochemical conditions, or lack scalability.^[^ ^43^, ^44^ ^]^

Herein, we report a facile near room‐temperature electric shock method to synthesize an efficient, stable, and inexpensive NiCoCuMoW multi‐element alloy on Ni foam within 400 s as a bifunctional electrocatalyst for both HOR and HER. This scalable method was conducted in mild and convenient conditions. Benefiting from the multi‐component synergy of MEA, 3D interconnected structure, and high conductivity, the resulting NiCoCuMoW showed remarkable intrinsic electrocatalytic performance and outstanding stability in alkaline electrolyte for both HOR and HER, rivaling the control samples and benchmark Pt/C catalyst, along with excellent CO tolerance. Density functional theory (DFT) calculations demonstrate that the alloying tailor the electronic structure of individual metals and provide multiple active sites for optimized hydrogen and hydroxyl intermediates adsorption, collaboratively accountable for the enhanced electrocatalytic HOR/HER activity.

## 2 RESULTS AND DISCUSSION

Among various metals, Ni,^[^ ^9^, ^16^, ^44^ ^]^ Co,^[^ ^19^, ^45^ ^]^ Cu,^[^ ^22^, ^46^ ^]^ Mo^[^ ^21^, ^47^ ^]^ and W^[^ ^22^, ^48^ ^]^ are widely studied for hydrogen electrocatalysts and exhibit low cost, high abundance, and decent activities. An ideal catalyst for HOR/HER should be active for both hydrogen and hydroxyl adsorption.^[^ ^49^ ^]^ Ni, Co, Cu are considered to exhibit favorable affinity for hydrogen binding, while Mo and W are supposed to be oxophilic which may be beneficial for hydroxyl adsorption.^[^ ^50^ ^]^ Thus, it's reasonable to adopt these elements to construct the MEA catalyst for improved HOR/HER. The MEA was fabricated through a pulse voltammetry method in a two‐electrode cell where a carbon rod serves as anode and a nickel foam (NF) as cathode. Electrolyte containing NiCl_2_, CoCl_2_, CuCl_2_, Na_2_WO_4_, and Na_2_MoO_4_ was maintained at 50°C with magnetic stirring at a speed of 500 rpm. The pulse voltage is set to be −6 V and operated at a duty cycle of 0.2 s (on)/0.8 s (off) for 400 s (see Experimental Section for more details).

**FIGURE 1:**
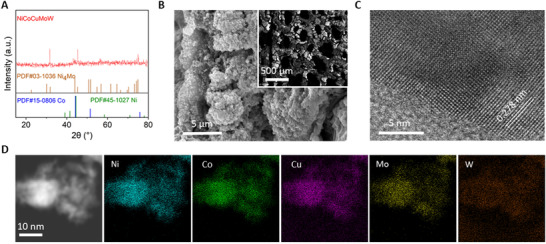
(A) X‐ray diffraction (XRD) pattern, (B) scanning electron microscopy (SEM) image, (C) high resolution transmission electron microscopy (HR‐TEM) image, and (D) TEM‐EDS (energy dispersive spectrometer) elemental mapping of the as‐prepared NiCoCuMoW electrocatalyst

The X‐ray diffraction (XRD) pattern (Figure 1A) of the resulting NiCoCuMoW demonstrated three diffraction peaks at 2*θ* = 31.66°, 45.44°, and 75.44°, which are different from those for standard metallic Ni, Co or Ni_4_Mo alloy. These peaks mismatching suggested the formation of new and pure alloy phase and further analysis revealed the formation of a body‐centered cubic structure (BCC) crystal, similar to reported MEA.^[^ ^37^, ^39^, ^40^ ^]^ The morphology of the NiCoCuMoW was then characterized by scanning electron microscopy (SEM) and high resolution transmission electron microscopy (HR‐TEM). The SEM image (Figure 1B) of the NiCoCuMoW electrocatalyst revealed the maintenance of 3D porosity structure of NF (inset of Figure 1B and Figure S1), and the presence of mainly coarse stacked particles over the skeleton surface. Low‐magnified TEM image (Figure S2) further showed its sheet‐stacked structure. Also, the HR‐TEM image (Figure 1C and Figure S2) revealed a lattice fringe of 0.278 nm, corresponding to the (100) planes of the BCC structure. Elemental mapping images suggested the uniform distribution of Ni, Co, Cu, Mo, and W throughout the whole sample (Figure 1D), in line with the SEM‐energy dispersive spectrometer (EDS) results (Figure S3). These results collectively verified the successful alloying of these metallic elements in NiCoCuMoW electrocatalyst.

**FIGURE 2:**
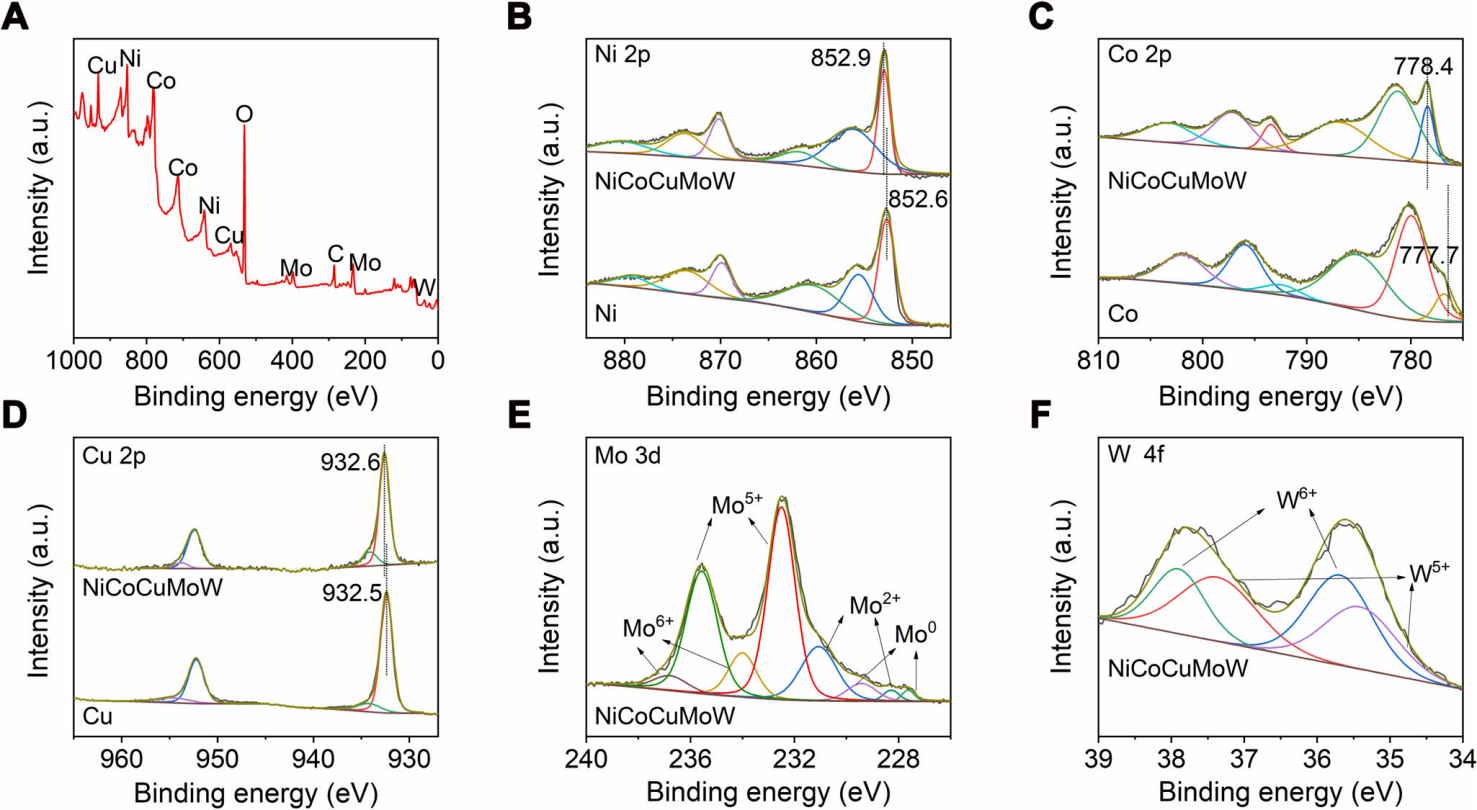
(A) X‐ray photoelectron spectroscopy (XPS) survey spectra of the as‐prepared NiCoCuMoW. High resolution XPS spectra of (B) Ni 2p, (C) Co 2p, (D) Cu 2p for the as‐prepared NiCoCuMoW and the corresponding monometallic controls. High resolution XPS spectra of (E) Mo 3d and (F) W 4f for the as‐prepared NiCoCuMoW

The surface elements and their valence states of NiCoCuMoW MEA were further probed by X‐ray photoelectron spectroscopy (XPS). Consistent with the elemental mapping and EDS results, the XPS survey spectra of the NiCoCuMoW (Figure 2A) showed the coexistence of Ni, Co, Cu, Mo, and W, plus O. High resolution XPS spectra for all the metals (Figure 2B–F) revealed their more or less oxidation due to exposure in air. For instance, the high resolution Ni 2p spectra shown in Figure 2B can be deconvoluted into six peaks assignable to metallic Ni (852.9 eV for Ni 2p_3/2_ and 870.2 eV for Ni 2p_1/2_) and Ni^2+^ (856.0 eV for Ni^2+^ 2p_3/2_ and 873.6 eV for Ni^2+^ 2p_1/2_). Similarly, the deconvolution of Co 2p XPS spectra (Figure 2C) suggested the coexistence of metallic and oxidation states (778.4, 793.4 eV for metallic Co and 781.2, 797.1 eV for Co^2+^). In terms of Cu, only a small part of Cu was oxidized and the dominant peaks can be assigned to the metallic Cu^0^ (932.7 eV for 2p_3/2_ and 952.3 eV for 2p_1/2_), as displayed in Figure 2D. For Mo 3d (Figure 2E) and W 4f (Figure 2F), most of the peaks can be assigned to the oxidative states of the corresponding metals. The similar oxidation of the as‐prepared MEA can also be found in previous reports.^[^ ^32^, ^51^, ^52^ ^]^ Notably, all the deconvoluted metal 2p spectra in NiCoCuMoW showed more or less shifting compared with those in monometallic Ni, Co, and Cu, suggesting the presence of strong electron coupling and transfer in the MEA system.^[^ ^53^ ^]^ This altering in electronic structure offered the possibility to tailor the surface charge state of multiple active sites in NiCoCuMoW MEA for optimized intermediates adsorption during the HOR/HER processes.

**FIGURE 3:**
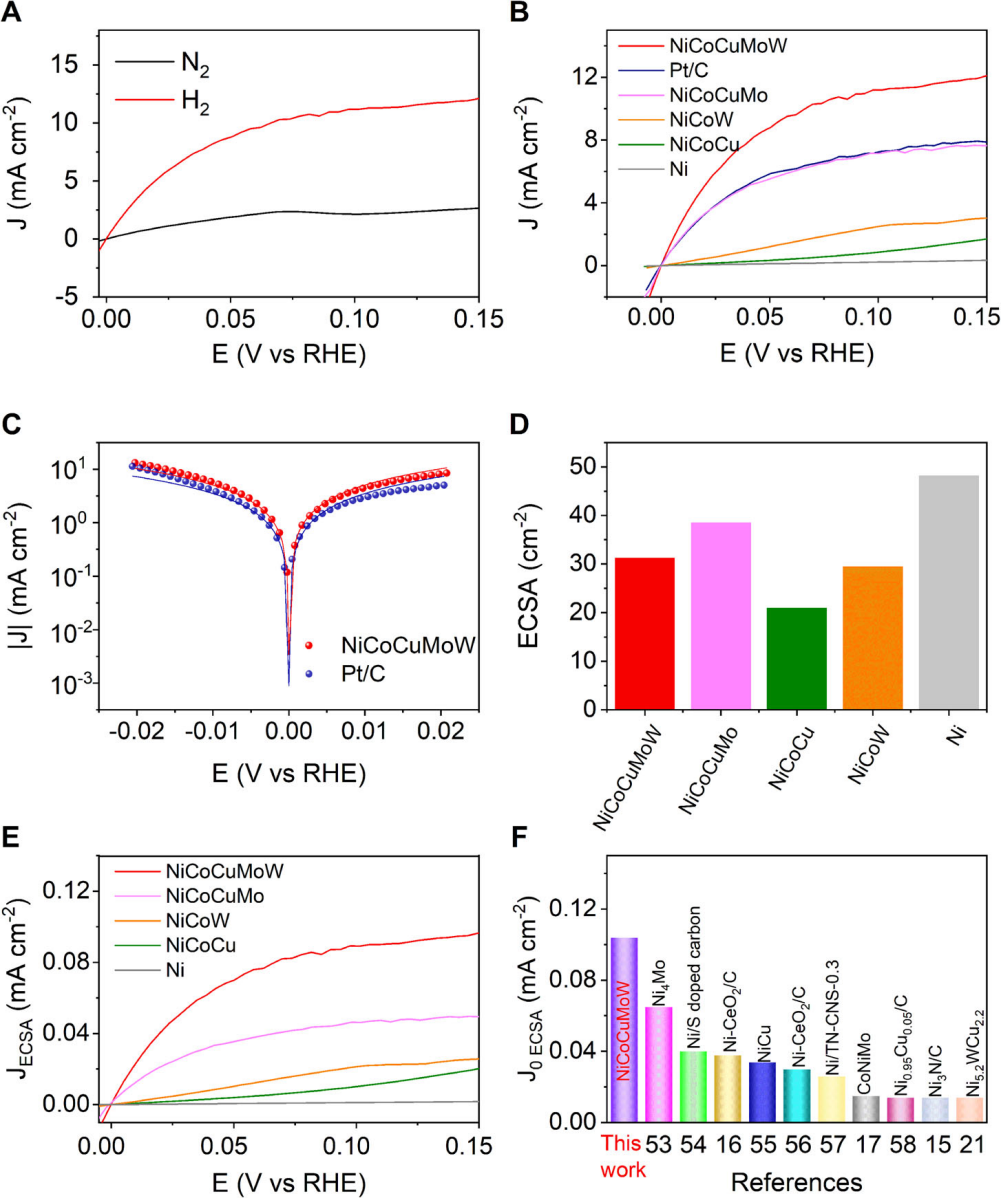
(A) Comparison of the SCV curves of NiCoCuMoW in H_2_‐saturated and N_2_‐saturated 1.0 m KOH. (B) The HOR performances of NiCoCuMoW, Pt/C, and control samples with fewer elements measured in H_2_‐saturated 1.0 M KOH with the SCV method. (C) HER/HOR Tafel plots of kinetic current density of NiCoCuMoW and Pt/C in H_2_‐saturated 1.0 M KOH, the points are obtained from SCV curves while the lines indicate the Butler–Volmer fitting. (D) Comparison of the calculated ECSA measured by CV method with the assumption that the *C*_dl_ of a flat smooth surface equals to 11 µF cm^−2^. (E) The SCV curves of NiCoCuMoW and control samples with fewer elements normalized by ECSA. (F) Comparison of ECSA‐normalized exchange current density of NiCoCuMoW and the state‐of‐the‐art nonprecious electrocatalysts

The HOR activity of the resulting NiCoCuMoW was first examined in a standard three‐electrode system with N_2_ or H_2_‐saturated 1 M KOH solution using a steady‐state staircase voltammetry (SCV) method (see Experimental Section), which leaves sufficient time between every two samplings, and thus allows the adequate transfer of hydrogen molecules and minimizes the capacitive current background.^[^ ^54^ ^]^ As shown in Figure 3A, a noticeable current density can be observed within H_2_‐saturated but not N_2_‐saturated KOH solution, confirming the pronounced electrocatalytic activity of NiCoCuMoW for HOR. Note that the NF substrate showed negligible current density (Figure S4). To highlight the critical role of multi‐element alloying, a series of control samples like monometallic Ni, Co, and Cu, bimetallic NiMo and MoW, trimetallic NiCoW and NiCoCu, and quadmetallic NiCoCuMo were prepared and tested under the same conditions for comparison. As depicted in Figure 3B and Figure S4A, the SCV curves of both monometallic Ni (Figure 3B), Co, Cu, and bimetallic NiMo and MoW (Figure S4A) demonstrated low current densities under the investigated potential windows (<0.4 mA cm^−2^), implying the poor HOR activities. Alloying Ni, Co with W or Cu resulted in observable increase of current density (∼2.2 mA cm^−2^ at 0.10 V versus reversible hydrogen electrode (RHE) for NiCoW and ∼0.9 mA cm^−2^ for NiCoCu), indicative of their decent HOR activities. Further alloying Ni, Co, Cu, and Mo, the HOR current density of the resulting NiCoCuMo electrocatalyst increased observably, achieving ∼7.1 mA cm^−2^ at 0.10 V versus RHE. Noticeably, the HOR current density at 0.10 V versus RHE can be further increased to ∼11.2 mA cm^−2^ for our resulting NiCoCuMoW MEA electrocatalyst. The commercial Pt/C catalyst was also loaded on NF for HOR testing and the optimized mass loading was 1.5 mg cm^−2^ (Figure S5). Figure 3B exactly revealed that the current density of as‐prepared NiCoCuMoW surpassed that of Pt/C at the whole potential range from 0 to 0.15 V versus RHE. For example, the current density of NiCoCuMoW soared up in a faster manner than Pt/C at the low potentials from 0 to 0.10 V versus RHE. At the potentials higher than 0.10 V, the current densities reached the plateaus and significantly higher current densities can be observed for NiCoCuMoW relative to Pt/C. Specifically, the current densities were ∼11.2 mA cm^−2^ for NiCoCuMoW and ∼ 7.2 mA cm^–2^ for Pt/C at 0.10 V versus RHE. We also emphasize that this value is even higher than most reported nonprecious HOR electrocatalysts including Ni_3_Ni/Ni/NF (∼7 mA cm^−2^)^[^ ^54^ ^]^ and PS‐MoNi/NF (∼ 4.5 mA cm^−2^).^[^ ^55^ ^]^ The high exchange current density (J_o_) further verified the superior HOR activity of NiCoCuMoW (Figure 3C). By fitting the SCV curves under micro‐polarization regions to the Butler–Volmer equation, the geometric J_o_ of NiCoCuMoW was determined to be 13.05 mA cm^−2^, largely exceeding that of the Pt/C catalyst (8.89 mA cm^−2^).

To get deeper insight into the inherent activity of the NiCoCuMoW electrocatalyst, we then examined the conductivity via electrochemical impedance spectroscopy (EIS) and the electrochemically active surface area (ECSA). As depicted in Figure S6, the Nyquist plots at 0.10 V versus RHE showed that the intercepts at high frequencies for NiCoCuMoW, NiCoCuMo, and Pt/C are similar and quite small, indicating their comparable conductivity. Besides, the ECSA measurement was completed by cyclic voltammetry (CV) in a region from −0.15 V to −0.05 V versus the open circuit potential (OCP) where no Faradaic process would happen.^[^ ^56^ ^]^ Supposing that the plate capacitance per unit area for a material is a constant value, then the electric double layer capacitance (*C*_dl_) is positively correlated with the ECSA, where the *C*_dl_ can be derived from the CV plots with different scan rates (Figure S7).^[^ ^56^ ^]^ Assuming the *C*_dl_ of a flat smooth surface is 11 µF cm^−2^, the ECSA of NiCoCuMoW was calculated to be 31.35 cm^2^, comparable to 38.64 cm^2^ for NiCoCuMo, 21.02 cm^2^ for NiCoCu, 29.57 cm^2^ for NiCoW and 48.34 cm^2^ for Ni (Figure 3D and Table S1). With these ECSA data in hand, their apparent current densities can be normalized to ECSA, and the ECSA‐normalized SCV curves for diverse electrocatalysts can be obtained (Figure 3E). Although the calculated ECSA of the NiCoCuMoW was not the largest, the NiCoCuMoW still demonstrated the most superior intrinsic HOR activity, as revealed by the highest ECSA‐normalized current densities under the applied potential range among the nonprecious electrocatalysts (Figure 3E). Furthermore, we also compared the ECSA normalized exchange current density (*J*_0 ECSA_) of NiCoCuMoW and the state‐of‐the‐art nonprecious HOR electrocatalysts. The *J*_0 ECSA_ getting from Butler–Volmer equation fitting was calculated to be 0.104 mA ${\mathrm{cm}}_{\mathrm{ECSA}}^{-2}$for NiCoCuMoW, in line with the results from linearly fitting in the micro‐polarization region (0.101 mA ${\mathrm{cm}}_{\mathrm{ECSA}}^{-2}$, Figure S8). This value outperformed most of the reported nonprecious HOR catalysts like Ni‐CeO_2_/C (0.038 mA${\mathrm{cm}}_{\mathrm{ECSA}}^{-2}$),^[^ ^17^ ^]^ Ni/NiO/C (0.026 mA ${\mathrm{cm}}_{\mathrm{ECSA}}^{-2}$),^[^ ^57^ ^]^ and Ni/S–doped carbon (0.0402 mA ${\mathrm{cm}}_{\mathrm{ECSA}}^{-2}$)^[^ ^58^ ^]^ (Figure 3F). More detailed information for this comparison^[^ ^59^, ^60^, ^61^ ^]^ could be found in Table S2.

**FIGURE 4:**
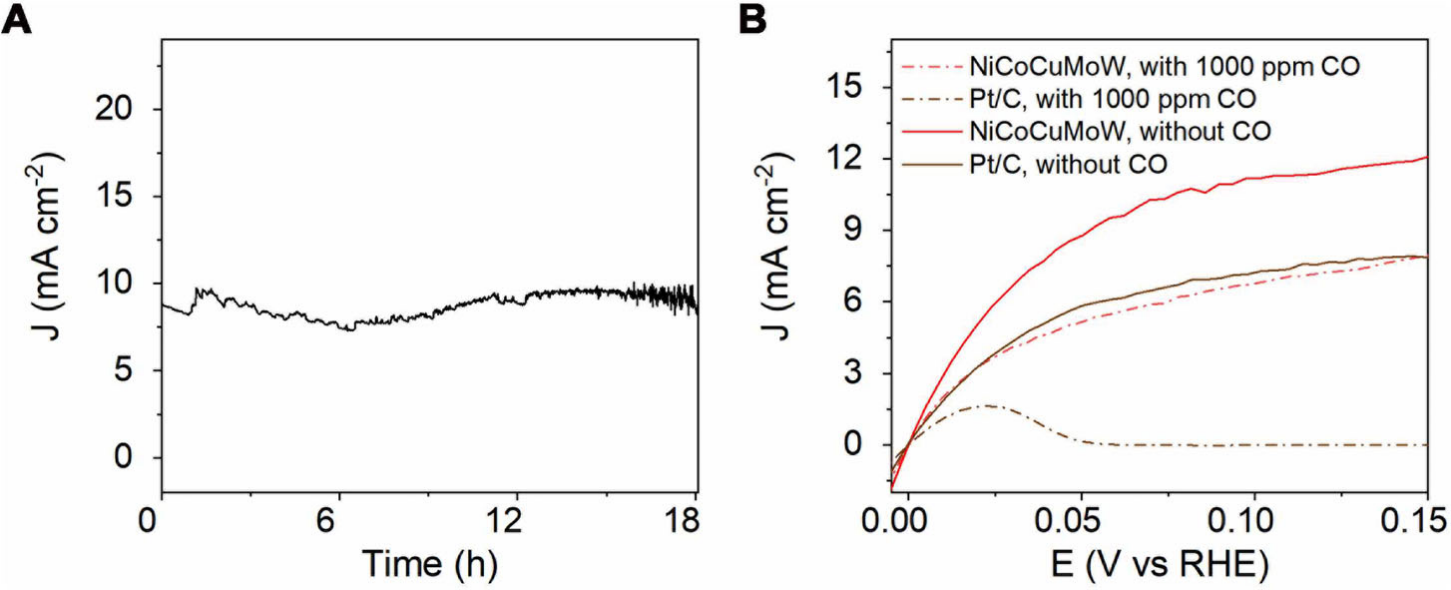
(A) Chronoamperometry curve of the NiCoCuMoW in H_2_‐saturated 1.0 M KOH measured at 0.13 V versus RHE. (B) Comparison of the steady‐state staircase voltammetry (SCV) curves of NiCoCuMoW and Pt/C in 1.0 M KOH saturated with H_2_ (solid lines) or H_2_ with 1000 ppm CO (dashed lines)

Besides high HOR activity, the NiCoCuMoW also exhibited robust long‐term electrochemical stability (Figure 4A). As plotted in Figure 4A, a chronoamperometry experiment of NiCoCuMoW was conducted at 0.13 V versus RHE in H_2_‐saturated 1.0 M KOH and a stable current density of ∼10 mA cm^−2^ was maintained during the 18 h HOR process. The fluctuation of the curve is ascribed to the bubbling of H_2_ and the stirring of a magnetic stirrer. For a practical application of the fuel cell, H_2_ feeding gas is commonly produced from steam reforming, the product of which often contains CO impurity in the final products, an ideal HOR electrocatalyst therefore should feature high tolerance to CO impurity. However, Pt‐based HOR electrocatalysts are highly sensitive to CO and a CO concentration as low as 100 ppm could lead to a considerable drop in HOR performance.^[^ ^60^, ^62^ ^]^ CO poisoning tolerance testing for our NiCoCuMoW MEA and Pt/C was conducted using a SCV method wherein the gas feeding contains 1000 ppm CO (V/V). As demonstrated in Figure 4B, both Pt/C and NiCoCuMoW suffered from more or less CO poisoning and exhibited declining performances. For Pt/C benchmark, the current density reached a peak value of 1.7 mA cm^−2^ at 23 mV versus RHE, which is less than half of the value measured in pure H_2_. The CO poisoning got more severe at high potentials and deactivated completely upon the potentials beyond 50 mV versus RHE. This might be explained by the long‐term interval (30 s for every 4 mV) during SCV tests, which enabled the sufficient mass transfer of H_2_, and at the same time, resulted in the accumulation of CO. For the NiCoCuMoW, although it was also poisoned by CO, its reduced current density was still comparative to Pt/C under pure H_2_ feeding. The CO tolerance might be originated from the multi‐active site nature of NiCoCuMoW, some of which were poisoned by CO while others remained active and finally endowed its high CO tolerance. This, once again, proved the superiority of the multi‐element strategy.

**FIGURE 5:**
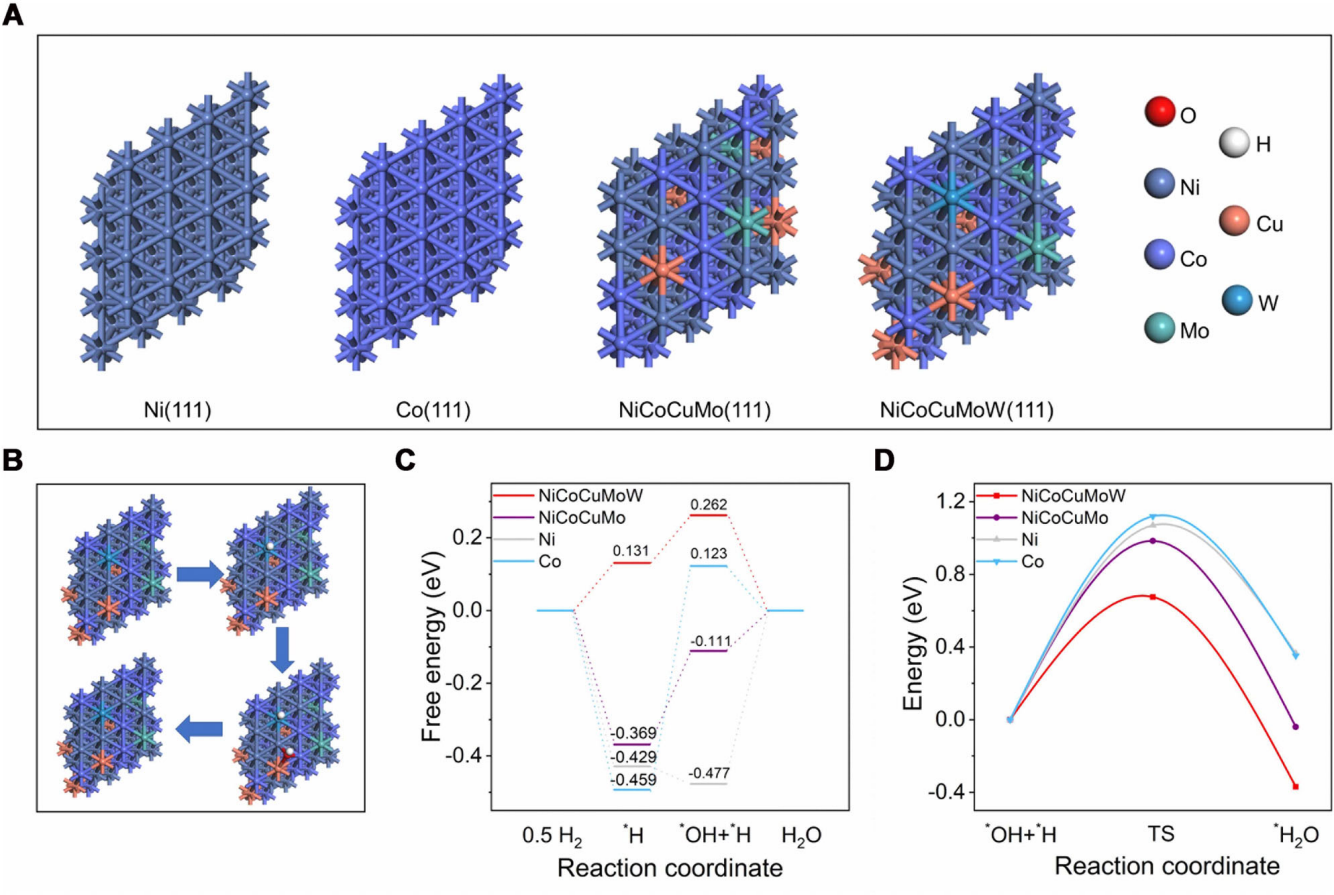
(A) Structural models for metallic Ni, Co, quaternary NiCoCuMo, and NiCoCuMoW. (B) Surface structural model of the various reaction species along the alkaline HOR pathway on NiCoCuMoW. (C) Gibbs free‐energy diagrams of alkaline HOR pathway on Ni, Co, NiCoCuMo, and NiCoCuMoW. (D) Reaction energy barrier diagrams of the alkaline HOR process from IS and TS to FS on Ni, Co, NiCoCuMo, and NiCoCuMoW

To get a deeper understanding of the mechanism responsible for the superb HOR activity of NiCoCuMoW, we performed Vien DFT calculations with Vienna Ab‐initio Simulation Package (VASP) under the Projected Augmented Wave (PAW) and the function of Perderw, Burke and Ernzerhof (PBE).^[^ ^63^, ^64^ ^]^ In accordance with the XRD results, (111) plane, the most exposed lowest‐energy facet with/without random elements distribution were simplified as models for calculations (Figure 5A). In a typical alkaline HOR process, H_2_ would first be adsorbed and dissociated into adsorbed H intermediates (*H) which then react with adsorbed OH species to generate H_2_O (Figure 5B and Figures S9–S12), such that not only the H binding energy (HBE) but also the OH binding energy (OHBE) account for HOR activity,^[^ ^22^ ^]^ which should be optimal values for neither too strong nor too weak binding. As shown in Figure 5C, the HBEs for Co(111), Ni(111), and NiCoCuMo(111) were calculated to be −0.459, −0.429, and −0.369 eV, respectively, under the equilibrium potential, indicative of too strong binding of H.^[^ ^22^ ^]^ The OHBE for Co(111) and NiCoCuMo(111) were 0.582 and 0.258 eV, suggesting the too weak OH binding. Although the OHBE for Ni(111) was suitable (−0.018 eV), the strong H binding over Ni(111) gave rise to the difficulty of combining *H and *OH to form H_2_O (rate‐limiting step) and a high free energy up‐hill (0.477 eV) can be obtained. In contrast, both the HBE and OHBE for our NiCoCuMoW(111) were quite similar and the biggest free energy up‐hill was only 0.131 eV, significantly lower than 0.582, 0.477, and 0.258 eV for Co(111), Ni(111), and NiCoCuMo(111) control samples, respectively. Therefore, according to the thermodynamic results, the NiCoCuMoW should exhibit the greatest active HOR activity.

Kinetics is also critical for alkaline HOR apart from thermodynamics.^[^ ^65^ ^]^ Therefore, the energy barriers for the whole HOR from the initial state (IS) and the transition state (TS) to the final state (FS) over these investigated nonprecious electrocatalysts were also calculated^[^ ^66^ ^]^ and the results (Figure 5D) showed that the energy barrier for NiCoCuMoW is 0.675 eV, much lower than 1.120 eV for Co, 1.070 eV for Ni and 0.985 eV for NiCoCuMo. Taken together, these experimental and DFT results clearly illustrated that alloying Ni, Co, Cu, Mo, and W can tune the electronic structure of separate metals and may provide multiple active sites to optimize the intermediate's adsorption during the HOR processes, collaboratively resulting in enhanced activity.

**FIGURE 6:**
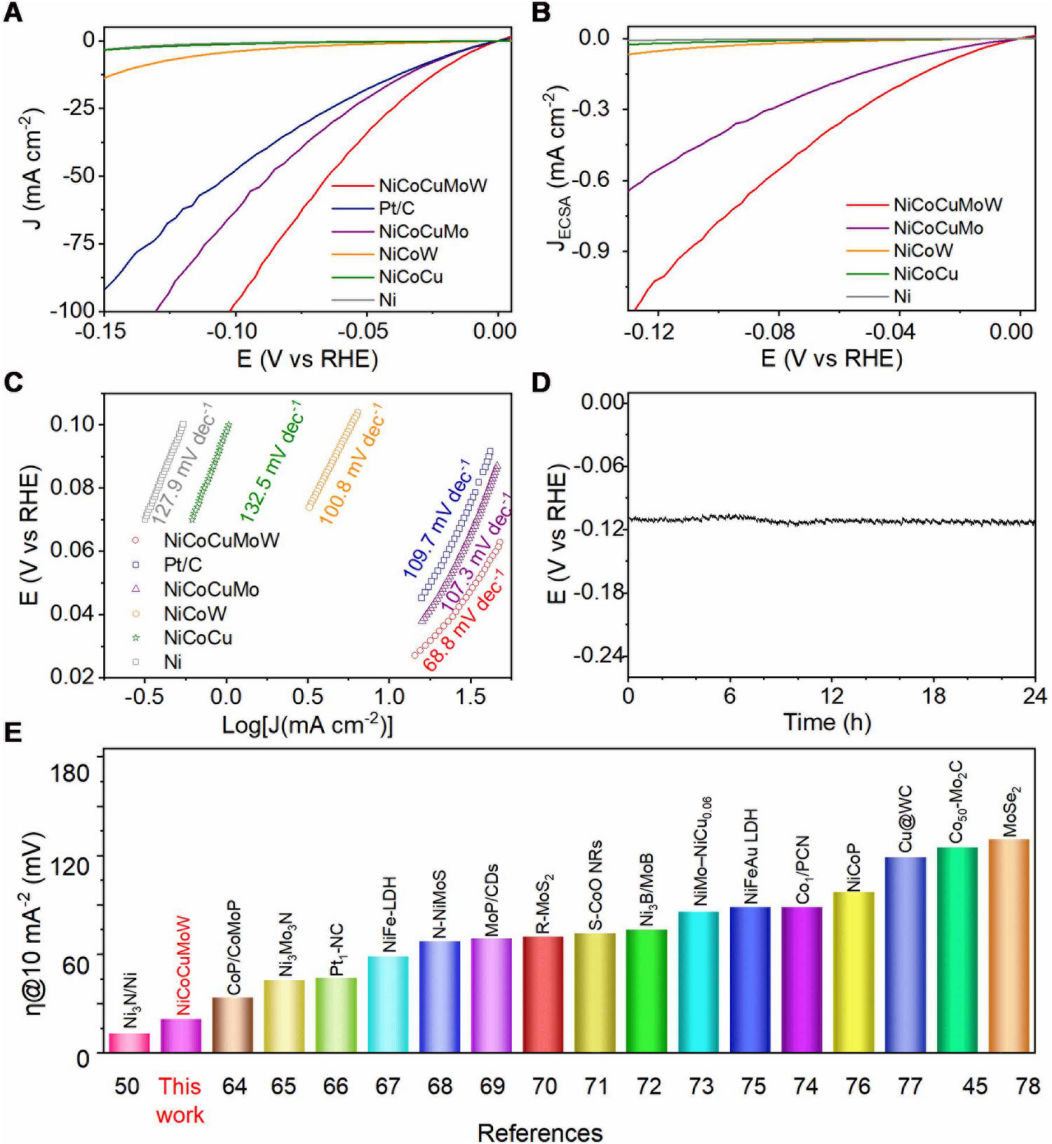
(A) Hydrogen evolution reaction (HER) polarization curves of NiCoCuMoW, Pt/C, and control samples with fewer elements measured in H_2_‐saturated 1.0 M KOH measured by the steady‐state staircase voltammetry (SCV) method. (B) ECSA‐normalized HER performance comparison of the NiCoCuMoW and control samples with fewer elements. (C) Tafel analysis for NiCoCuMoW, Pt/C, and control samples with fewer elements in H_2_‐saturated 1.0 M KOH. (D) Chronopotentiometry curves of NiCoCuMoW in H_2_‐saturated 1.0 M KOH measured at a current density of 100 mA cm^−2^. (E) Comparison of overpotentials (*η*) at 10 mA cm^−2^ for NiCoCuMoW and the state‐of‐the‐art nonprecious electrocatalysts

In parallel with HOR in a fuel cell for hydrogen utilization, its reverse reaction, HER in water splitting for hydrogen generation is equally important.^[^ ^1^, ^6^ ^]^ The good HOR performance of the as‐prepared NiCoCuMoW inspired us to determine its HER activity in the same electrolyte (1.0 m KOH). We compared the HER polarization curves using SCV and traditional linear sweep voltammetry (LSV) methods, and found that the influence of capacitive current

cannot be ignored (Figure S13). Such that the SCV method instead of traditional LSV method was adopted by us to estimate the HER performance of all the electrocatalysts accurately. As shown in Figure 6A, the overpotential at 10 mA cm^−2^ (*η*_10_) for the NiCoCuMoW was only 21 mV, much lower than those of NiCoCuMo (30 mV), NiCoW (138 mV), NiCoCu (>138 mV), and even Pt/C (35 mV). Actually, the *η*_10_ of 21 mV was lower than most reported catalysts (Figure 6E and Table S3),^[^ ^67^, ^68^, ^69^, ^70^, ^71^, ^72^, ^73^, ^74^, ^75^, ^76^, ^77^, ^78^, ^79^, ^80^, ^81^ ^]^ such as Co_1_/PCN (89 mV),^[^ ^81^ ^]^ Pt_1_‐NC (46 mV),^[^ ^69^ ^]^ NiFe‐LDH (59 mV),^[^ ^70^ ^]^ and S‐CoO NRs (73 mV).^[^ ^74^ ^]^ Note that the HER currents of Ni foam, metallic Ni, Co, NiMo, and MoW were nearly negligible (Figure 6A and Figure S4B). When normalized by ECSA, the ordering of intrinsic activity remained unchanged, emphasizing the critical role of multi‐element alloying (Figure 6B). Noticeably, the HER current density of NiCoCuMoW also largely exceeded that of Pt/C benchmark electrode under all the potential regions. Additionally, the smaller Tafel slope (63.7 mV dec^−1^) in comparison to those of all control samples with fewer elements and Pt/C (109.7 mV dec^−1^) suggested the faster HER kinetics on the NiCoCuMoW (Figure 6C). Besides excellent HER activity, the NiCoCuMoW also maintained the overpotential of 108 mV at 100 mA cm^−2^ during the 24 h‐chronopotentiometry tests, demonstrating the superb stability (Figure 6D).

## 3 CONCLUSION

In summary, we have synthesized a MEA of NiCoCuMoW through a facile electroshock method under near room‐temperature within 400s. The resulting MEA electrocatalyst delivers a high current density of ∼ 11.2 mA cm^−2^ at the overpotential of 100 mV for HOR in 1.0 m KOH which is even higher than the commercial Pt/C catalyst, along with long‐term stability and high CO tolerance. The high exchange current density of 0.104 mA ${\mathrm{cm}}_{\mathrm{ECSA}}^{-2}$ is also one of the highest among the state‐of‐the‐art nonprecious HOR electrocatalysts. In addition, an overpotential of only 21 mV at a current density of 10 mA cm^−2^ can be obtained for HER in the same electrolyte, lower than most reports. Control experiments and DFT calculations indicate that the superb HOR and HER activity of NiCoCuMoW could be explained by the optimized binding of critical intermediates including hydrogen and hydroxyl species due to the multi‐component synergy of MEA which tunes the electronic structure of individual metals and provides multiple active sites. We envisage that such a facile and effective electroshock method can be extended to synthesize diverse multi‐element alloys or oxides for many other catalytic applications.

## 4 EXPERIMENTAL SECTION

### 4.1 Preparation of NiCoCuMoW MEA and control samples

The NiCoCuMoW was fabricated by an electroshock method in an electrochemical working station (Gamry Interface 5000). The electrolyte (36 ml) was prepared by mixing 0.3 M CoCl_2_, 0.5 M NiCl_2_, 0.004 M CuCl_2_, 0.045 M Na_2_MoO_4_, 0.045 M Na_2_WO_4_, 0.4 M Na_3_C_6_H_5_O_7_, and 0.2 g L^–1^ sodium dodecyl sulfate (SDS) in ultrapure water and was stirred at 500 rpm at 50°C. A two‐electrode cell was fabricated with a carbon rod as the anode and a piece of nickel foam (NF) as the cathode. The optimal electric shock time was 400 s (Figure S14). After the electroshock, the plated cathodic electrode was rinsed with water and dried with a rubber suction bulb.

A series of control samples including NiCoCuMo, NiCoW, NiMo, NiCoCu, and MoW was prepared using the same method of NiCoCuMoW, except the absence of corresponding salts in the electrolyte. For the Pt/C control, 2 mg 20% Pt/C was mixed with a solution containing 550 µl ethanol, 400 µl DI water, and 50 µl 5 wt% Nafion solution and then the solution was ultrasonicated for 30 min to form a homogenous ink suspension. A certain number of the solution drops were cast onto the NF to get different loading amounts of Pt/C (1.0, 1.5, and 2.0 mg cm^−2^).

### 4.2 Electrochemical measurement

The HER and HOR performances were measured using a SCV method in CHI 760E electrochemical workstation with a three‐electrode configuration. The potential increasement was set to 0.4 mV and step period was 30 s, which suggested that for every 30 s, the potential increases 0.4 mV, and the corresponding current is recorded. The as‐prepared monolithic NiCoCuMoW was directly used as the working electrode. A calibrated Ag/AgCl (saturated KCl) with salt bridge kit and a glassy carbon was chosen as the reference and counter electrode, respectively. 1 M KOH bubbled with H_2_ throughout the whole electrochemical experiments is used as the electrolyte for both HER and OER measurements. According to previous reports and our experiments (Figure S15), 1.0 M KOH is a more critical environment for HOR.^[^ ^61^ ^]^

Stability tests were conducted using chronoamperometry for HOR and chronopotentiometry for HER. The chronoamperometry was conducted in H_2_‐saturated 1.0 M KOH measured at 0.13 V versus RHE. The chronopotentiometry was conducted in H_2_‐saturated 1.0 M KOH measured at 100 mA cm^−2^.

The EIS measurement was performed at 0.1 V and with a frequency range from 0.1 Hz to 10 MHz. All potentials are reported versus RHE.

ECSA was measured by CV method. The CV curves were collected in a non‐Faradaic potential region from −0.15 to −0.05 V versus OCP at various scan rates ranging from 4 to 20 mV s^−1^ in CH_3_CN with 0.15 M NH_4_PF_6_. By plotting the difference between the anodic and cathodic current densities (Δ*J*) at −0.10 V versus OCP against the scan rate, a linear slope could be obtained. This linear slope is twice of the *C*_dl_. The ECSA can be calculated according to the following equation: ECSA = *C*_dl_/*C*_s_, where *C*_s_ is the specific capacitance of a flat smooth surface of the electrode material, which is assumed to be 11 µF cm^−2^ according to the literature report.^[^ ^56^ ^]^

The exchange current density (*j*_0_) can be obtained by Butler–Volmer method or micro‐polarization method. In the Butler–Volmer method, Butler–Volmer Equation was used to fit the relationship between kinetic current density(*j*_k_) and overpotential (*η*):

(1) $$j_{k}\;=\;j_{0}\;(e^{\frac{\alpha F}{\mathrm{RT}}\eta }-e^{\frac{\left(\alpha -1\right)F}{\mathrm{RT}}\eta })$$

where *α* is the charge transfer coefficient, *η* is the overpotential, R is the ideal gas constant (8.314 J mol^−1^ K^−1^), *T* is the experimental temperature (298 K), and F is the Faradaic constant (96,485 C mol^−1^). As for the micro‐polarization method, in the small potential window of the micro‐polarization region near the equilibrium potential (± 10 mV vs. RHE), the Butler–Volmer equation can be expanded by Taylor's formula in the micro‐polarization region near the equilibrium potential (± 10 mV vs. RHE), and simplified as equation:

(2) $$j=j_{0}\eta FRT$$

Therefore, *j*_0_ can be obtained from the slope of the linear fitting in the micro‐polarization region.

### 4.3 Density functional theory calculation

Spin‐polarized DFT calculations were performed using VASP under the PAW method. The exchange‐correlation energy was described by the function of PBE. For geometry optimizations, the cut‐off energy was set to 500 eV. A 3 × 3 × 1 Monkhorst–Pack grid was set to carry out surface calculations on all models. Vertical interactions in the z direction between slabs were avoided by setting a vacuum layer of 15 Å. The convergence criteria were set to be 10^−5^ eV for total energy and 0.02 eV Å^−1^ for force per atom. The van der Waals dispersion obtained via the D3 method of Grimme was considered in all calculations. CI‐NEB was applied to compute the decomposition barriers of H_2_O molecular to obtain the minimum energy path between the given initial and final positions.

## CONFLICT OF INTEREST

The authors declare no conflict of interest.

## Supporting information



Supporting information

## Data Availability

The data that support the findings of this study are available from the corresponding author upon reasonable request.
