# Supplementary material for: Electroshock synthesis of a bifunctional nonprecious multi‐element alloy for alkaline hydrogen oxidation and evolution
Source: Exploration (Beijing). 2022 Nov 24;2(6):20220024. doi: 10.1002/EXP.20220024 (PMC10190983; doi:10.1002/EXP.20220024)
Supplement: Supplementary file 1 — Supporting information [file EXP2-2-20220024-s001.docx]

**Supporting Information**

Electroshock Synthesis of a Bifunctional Nonprecious Multi-Element Alloy for Alkaline Hydrogen Oxidation and Evolution

Lijie Du, Hu Xiong, Hongcheng Lu, Li-Ming Yang, Rong-Zhen Liao, Bao Yu Xia and Bo You*


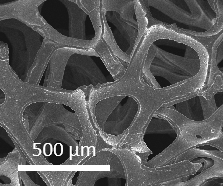


**Figure S1** SEM image of the Ni foam (NF) substrate.





**Figure S2** Low-magnified TEM image of the as-prepared NiCoCuMoW.


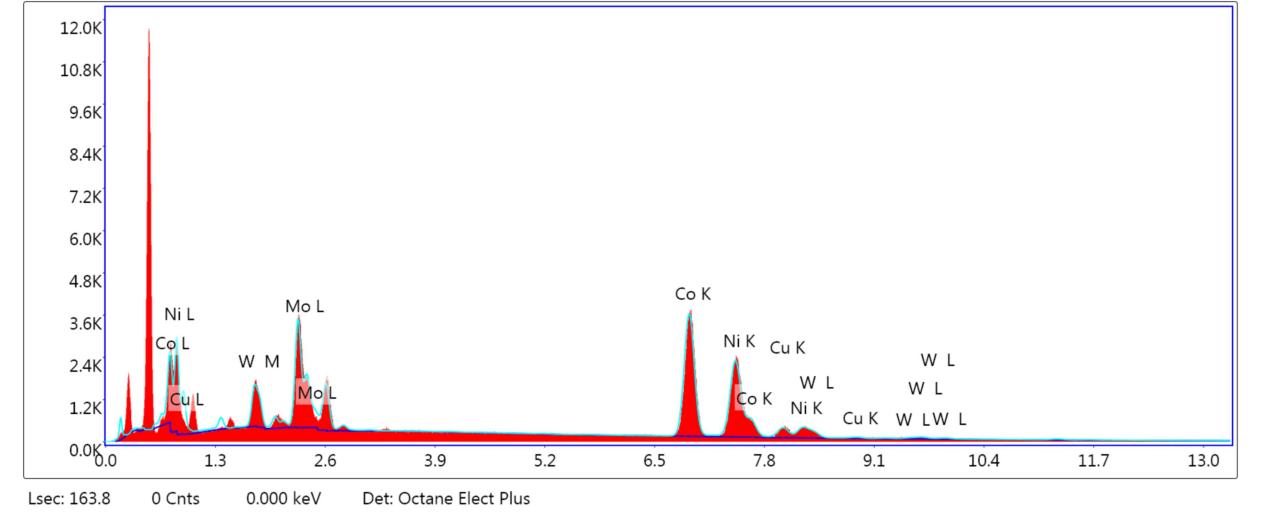


**Figure S3** SEM-EDS pattern of the as-prepared NiCoCuMoW.





**Figure S4** SCV curves of (A) HOR and (B) HER of NiMo, MoW and Ni foam (NF) measured in H_2_ saturated 1.0 M KOH.





**Figure S5** SCV curves of commercial Pt/C catalyst with different mass loading. The results show that bot the HER and HOR performances would not increase when the mass loading is higher than 1.5 mg cm^-2^.





**Figure S6** Nyquist plots at 0.1 V vs RHE for NiCoCuMoW, Pt/C and control samples including NiCoCuMo, NiCoW, NiCoCu and Ni.





**Figure S7** Electrochemically active surface area (ECSA) measurements with CV method. Left: the CV curves were collected in a non-Faradaic potential region from −0.15 to −0.05 V vs open circuit potential (OCP) at various scan rates ranging from 4 to 20 mV s^-1^ in CH_3_CN with 0.15 M NH_4_PF_6_. Right: the linear fitting between scan rate and the difference between the anodic and cathodic current densities (ΔJ).

Table S1 Comparison of the calculated ECSAs for diverse electrocatalysts according to the results in Figure S7.

|  | NiCoCuMoW | NiCoCuMo | NiCoCu | NiCoW | Ni |
| --- | --- | --- | --- | --- | --- |
| Slope (mF cm^-2^) | 2.8 | 3.4 | 1.9 | 2.9 | 4.3 |
| ECSA (cm^-2^) | 31.35 | 38.64 | 21.02 | 29.57 | 48.34 |

.



**Figure S8** Linear fitting in the micro-polarization region between the potential and the current density. The fitting result indicates that the slope value is 3.92. Then the exchange current density can be calculated by multiply the value slope with RT/F, where R is the ideal gas constant (8.314 J mol^-1^ K^-1^), T is the experimental temperature (298 K), and F is the Faraday constant (96,485 C mol^-1^). The calculation result is 0.101 mA $\text{cm}_{\text{ECSA}}^{\text{-2}}$.

Table S2 Comparison of the electrocatalytic alkaline HOR performance of the NiCoCuMoW with those of reported nonprecious electrocatalysts

| Catalyst | Description | Exchange current density (mA $\text{cm}_{\text{ECSA}}^{\text{-2}}$) | Reference |
| --- | --- | --- | --- |
| NiCoCuMoW | Multi-element alloy containing Ni, Co, Cu, Mo, W. | 0.104 | This work |
| Ni-H_2_-NH_3_ | Ni_3_(BTC)_2_ pyrolyzed at 390°C within mixed H_2_/NH_3_/N_2_ gases | 0.070 | *Nat. Mater.,* 2022, 21, 804. |
| N-Ni | Nitrogen-inserted Ni nanosheets | 0.041 | *Energy Environ. Sci.,* 2022, 15, 1234. |
| Ni_5.2_WCu_2.2_ | Nickel-tungsten-copper alloy | 0.014 | *Nat. Commun.,* 2021, 12, 2686. |
| Ni_4_Mo | Interconnected alloy nanosheets | 0.065 | *Nat. Commun.,* 2020, 11, 4789. |
| Ni-CeO_2_/C | Ni/CeO_2_ heterostructures | 0.038 | *Angew. Chem. Int. Ed.,* 2019， 58, 14179. |
| Ni/NiO/C | Ni@NiO core-shell (20-40 nm) NPs in carbon framework | 0.026 | *Angew. Chem. Int. Ed.,* 2019，58, 10644. |
| Ni_3_N/C | ≈4.6 nm NPs | 0.014 | *Angew. Chem. Int. Ed.,* 2019, 58, 7445. |
| CoNiMo | CoNiMo deposited on a polycrystalline gold (Au) disk | 0.015 | *Energy Environ. Sci.,* 2014, 7, 1719. |
| NiCu | Ni–Cu binary alloy films | 0.034 | *ACS. Appl. Energy Mater.,* 2019, 2, 3160. |
| Ni_0.95_Cu_0.05_/C | ≈30 nm | 0.014 | *J. Electroanal. Chem.,* 2016, 783, 146. |
| Ni/S-C | Ni NPs (14-18 nm) supported on S-doped carbon | 0.0402 | *J. Mater. Chem. A,* 2019, 7, 10936. |
| Ni/TN-CNS | Ni NPs (10-15 nm) supported on N-doped carbon nanosheets | 0.03 | *Chem. Phys. Lett.,* 2019, 728, 19. |


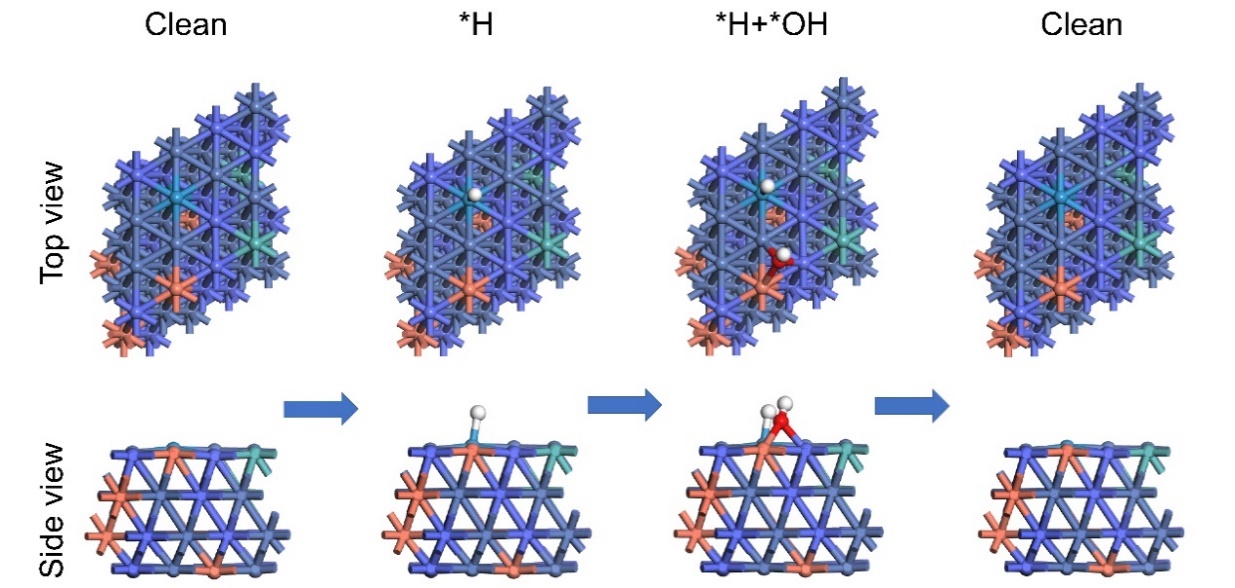


Figure S9 Simplified configurations of various reaction species along the reaction pathway on the surface of NiCoCuMoW.


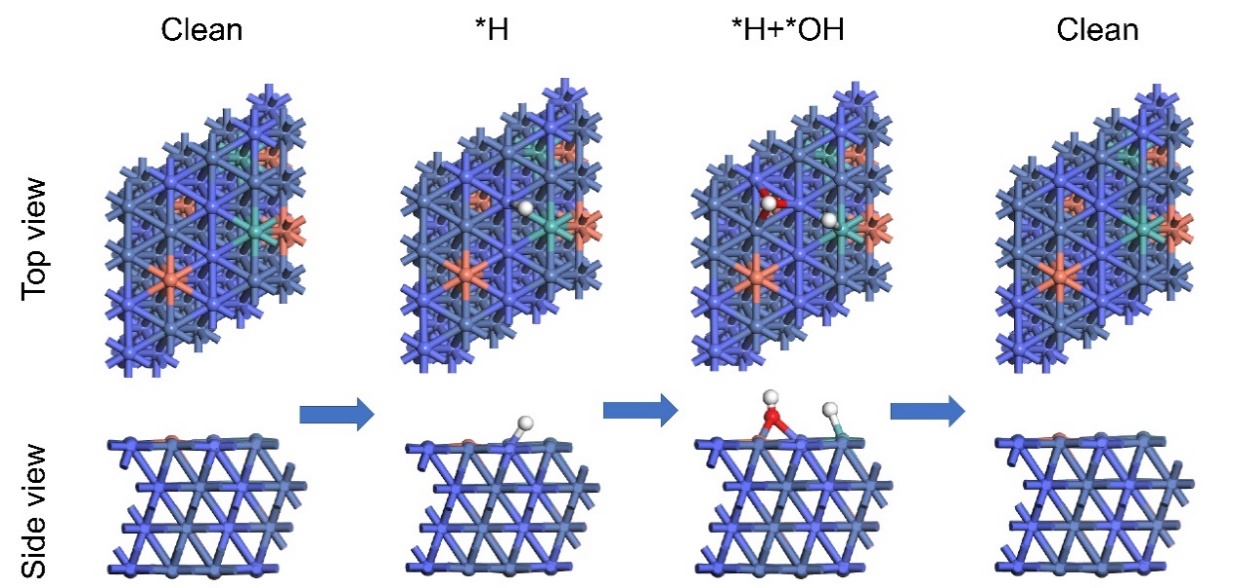


Figure S10 Simplified configurations of various reaction species along the reaction pathway on the surface of NiCoCuMo.


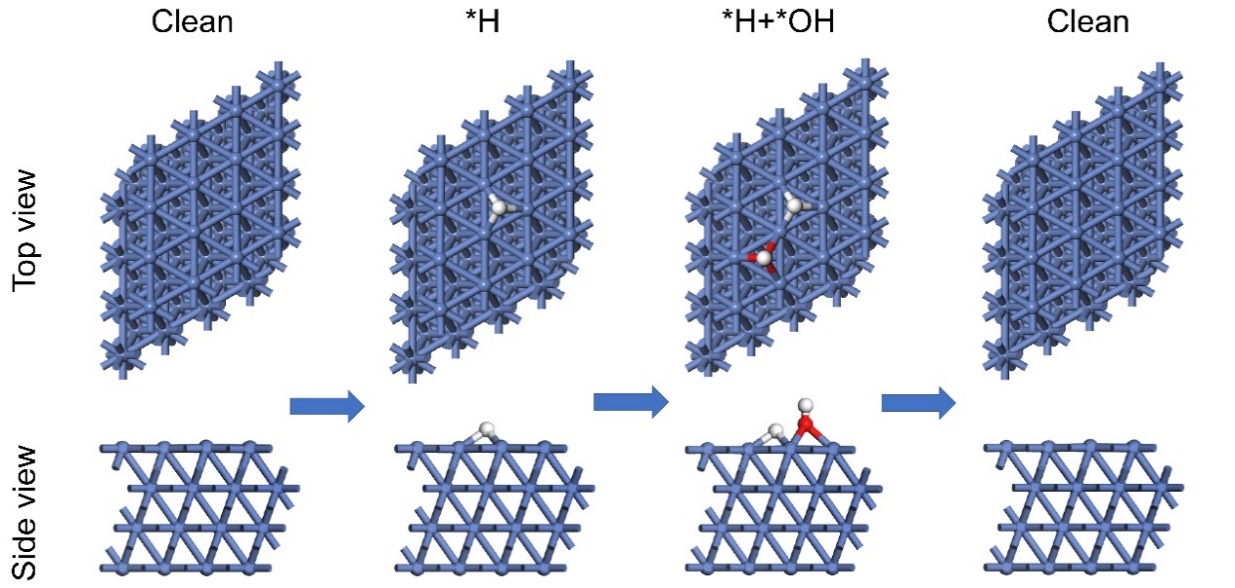


Figure S11 Simplified configurations of various reaction species along the reaction pathway on the surface of Ni.


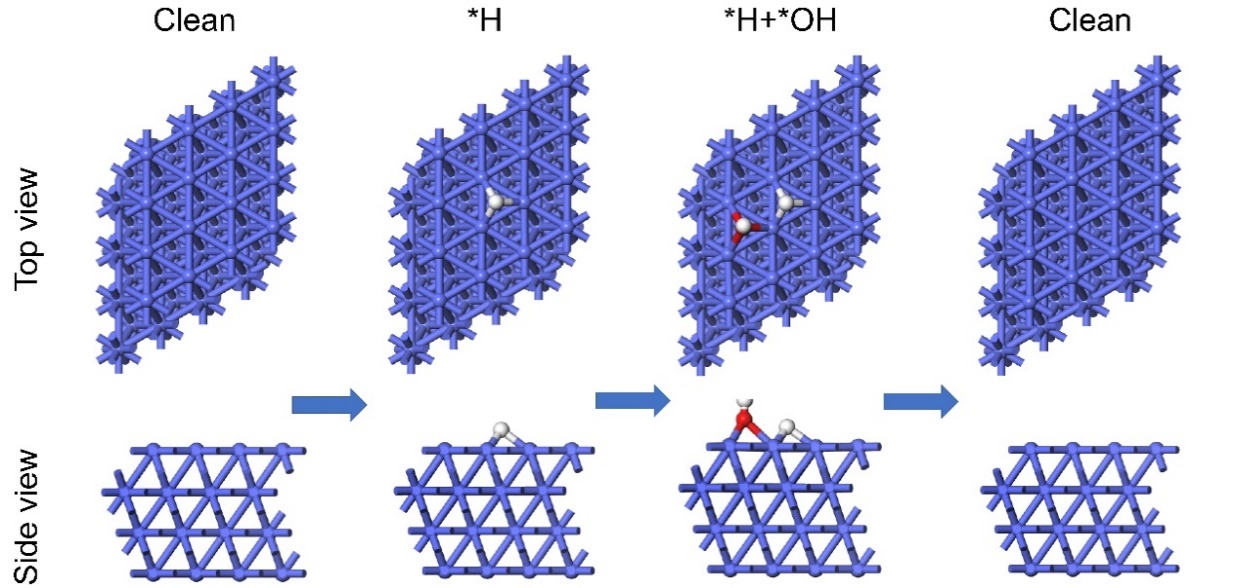


Figure S12 Simplified configurations of various reaction species along the reaction pathway on the surface of Co.





**Figure S13** HOR and HER performances of NiCoCuMoW measured by LSV and SCV. The scan rate of LSV is 2 mV s^-1^. The difference between them is attributed to the capacitance current.


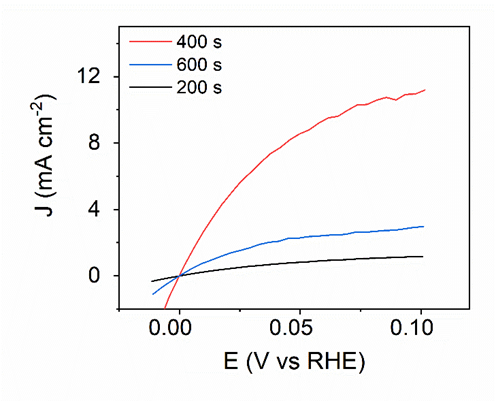


**Figure S14** The HOR performance of NiCoCuMoW with different deposition time.





**Figure S15** The HOR performances of the NiCoCuMoW in 0.1 M and 1.0 M KOH.

Table S3 Comparison of the electrocatalytic alkaline HER performance of the NiCoCuMoW with those of reported nonprecious electrocatalysts

| Catalysts | Description | η_10_ (mV) | Reference |
| --- | --- | --- | --- |
| NiCoCuMoW | Multi-element alloy containing Ni, Co, Cu, Mo, and W. | 21 | This work |
| Co_1_/PCN | Co_1_-N_4_ moiety in the framework of phosphorized carbon nitride | 89 | *Nat. Catal.,* **2019**, 2, 134. |
| R-MoS_2_ | MoS_2_ with multi-functional active sites | 71 | *Adv. Mater.,* 2018, 30, 1707105. |
| S-CoO NRs | O-vacancies on the surface of strained CoO nano ribbon | 73 | *Nat. Commun.,* 2017, 8, 1509. |
| NiFe-LDH | NiFe layered double hydroxide | 59 | *Energy Environ. Sci.,* 2019, 12 572. |
| Ni_3_N/Ni | Ni_3_N/Ni interface | 12 | *Nat. Commun.,* **2018**, 9, 4531. |
| N-NiMoS | Ni/CeO_2_ heterostructures | 68 | *Appl. Catal., B,* 2020, 276, 119137. |
| Cu@WC | Ni@NiO partial core-shell (20–40 nm) NPs in carbon framework | 119 | *Appl. Catal., B,* **2021**, 280 119451. |
| CoP/CoMoP | CoP nanowires array coupled with defective CoMoP nanosheets | 34 | *Nano Energy,* 2020, 68, 104332. |
| Ni_3_B/MoB | Ni_3_B/MoB heterostructure with abundant grain boundaries | 75 | *Chem. Eng. J.,* 2021, 405, 126977. |
| MoSe_2_ | 2-D MoSe_2_ | 130 | *ACS Nano,* 2020, 14, 6295. |
| Pt_1_-NC | Pt single atom dispersed in N doped carbon | 46 | *Nat. Commun.,* **2020**, 11, 1029. |
| MoP/CDs | N-doped, defect-containing, carbon-dots-loaded molybdenum phosphide | 70 | *Nano Energy,* 2020, 72, 104730. |
| Co_50_-Mo_2_C | Co-doped porous Mo_2_C | 125 | *Adv. Funct. Mater.,* 2020, 30, 2000561. |
| NiFeAu LDH | NiFe LDH by partially substituting Fe centers with Au | 89 | *Catal. Sci. Technol.,* 2020, 10, 4184. |
| NiMo-NiCu_0.06_ | NiMo-NiCu coatings with MoNi_4_ and NiCu phases | 86 | *ACS Appl. Mater. Interfaces,* 2020, 12, 17492. |
| Ni_3_Mo_3_N | N-doped carbon-coated porous Ni_3_Mo_3_N microrods | 44.6 | *Appl. Catal., B,* 2020, 272, 118956. |
| NiCoP | Self-supported NiCoP nano-leaves | 98 | *J. Energy Chem.,* 2020, 50, 395. |
| Pt-SAC-MoS_2_ | Single Pt atoms on MoS_2_ support | 29 | *Nat. Commun.,* 2021, 12, 3021. |
| NiS_2_/NiS_2_-NiS | NiS_2_ nanoflowers on heterogeneous NiS_2_-NiS foam | 55 | *Adv. Energy Mater.,* 2022, 12, 2103359. |
| FeP NFSLs | 2D FeP nanoframes superlattices | 71 | *Adv. Mater.,* 2022, 34, 2109145. |
